# Supplementary material for: Delivering clinical studies of exercise in the COVID-19 pandemic: challenges and adaptations using a feasibility trial of isometric exercise to treat hypertension as an exemplar
Source: BMJ Open. 2023 Mar 17;13(3):e068204. doi: 10.1136/bmjopen-2022-068204 (PMC10030278; doi:10.1136/bmjopen-2022-068204)
Supplement: Author's manuscript [file bmjopen-2022-068204.draft_revisions.pdf]

BMJ Open is committed to open peer review. As part of this commitment we make the peer review history of every article we publish publicly available.

When an article is published we post the peer reviewers' comments and the authors' responses online. We also post the versions of the paper that were used during peer review. These are the versions that the peer review comments apply to.

The versions of the paper that follow are the versions that were submitted during the peer review process. They are not the versions of record or the final published versions. They should not be cited or distributed as the published version of this manuscript.

BMJ Open is an open access journal and the full, final, typeset and author-corrected version of record of the manuscript is available on our site with no access controls, subscription charges or pay-per-view fees (http://bmjopen.bmj.com).

If you have any questions on BMJ Open's open peer review process please email info.bmjopen@bmj.com

BMJ Open

Delivering clinical studies of exercise in the COVID-19 pandemic: Challenges and adaptations using a feasibility trial of isometric exercise to treat hypertension as an exemplar.

Journal:	*BMJ Open*
Manuscript ID	bmjopen-2022-068204.R1
Article Type:	Communication
Date Submitted by the Author:	03-Dec-2022

Complete List of Authors:	Farmer, C; University of Kent, Centre for Health Services Studies; East Kent Hospitals University NHS Foundation Trust, Renal Department<br>Santer, Ellie; University of Kent, Centre for Health Services Studies<br>West, Alan; Public co-applicant<br>Darby, John; Public co-applicant<br>Rees-Roberts, Melanie; University of Kent, Centre for Health Services Studies<br>Doulton, Timothy; East Kent Hospitals University NHS Foundation Trust, Renal Department<br>MacInnes, Douglas; Canterbury Christ Church Univ, Facult yof Health and Wellbeing<br>O'Driscoll, Jamie; Canterbury Christ Church University, Sport, Exercise and Rehabilitation Sciences<br>Borthwick, Rachel; University of Kent Centre for Health Services Studies<br>Pellatt-Higgins, T; University of Kent, Centre for Health Services studies<br>Gousia, Katerina; University of Kent, Centre for Health Services Studies<br>Short, Vanessa; Newton Place Surgery<br>Swift, Pauline; Epsom and St Helier University Hospitals NHS Trust, Research and Development<br>Wiles, Jonathan; Canterbury Christ Church University, Sport, Exercise and Rehabilitation Sciences

**Primary Subject Heading**:	Communication
Secondary Subject Heading:	Cardiovascular medicine, Evidence based practice, Health services research
Keywords:	Clinical trials < THERAPEUTICS, Hypertension < CARDIOLOGY, SPORTS MEDICINE

SCHOLARONE™
Manuscripts

I, the Submitting Author has the right to grant and does grant on behalf of all authors of the Work (as defined in the below author licence), an exclusive licence and/or a non-exclusive licence for contributions from authors who are: i) UK Crown employees; ii) where BMJ has agreed a CC-BY licence shall apply, and/or iii) in accordance with the terms applicable for US Federal Government officers or employees acting as part of their official duties; on a worldwide, perpetual, irrevocable, royalty-free basis to BMJ Publishing Group Ltd ("BMJ") its licensees and where the relevant Journal is co-owned by BMJ to the co-owners of the Journal, to publish the Work in BMJ Open and any other BMJ products and to exploit all rights, as set out in our licence.

The Submitting Author accepts and understands that any supply made under these terms is made by BMJ to the Submitting Author unless you are acting as an employee on behalf of your employer or a postgraduate student of an affiliated institution which is paying any applicable article publishing charge ("APC") for Open Access articles. Where the Submitting Author wishes to make the Work available on an Open Access basis (and intends to pay the relevant APC), the terms of reuse of such Open Access shall be governed by a Creative Commons licence – details of these licences and which Creative Commons licence will apply to this Work are set out in our licence referred to above.

Other than as permitted in any relevant BMJ Author's Self Archiving Policies, I confirm this Work has not been accepted for publication elsewhere, is not being considered for publication elsewhere and does not duplicate material already published. I confirm all authors consent to publication of this Work and authorise the granting of this licence.

For peer review only

Delivering clinical studies of exercise in the COVID-19 pandemic: Challenges and adaptations using a feasibility trial of isometric exercise to treat hypertension as an exemplar.

Chris Farmer[1], Ellie Santer[1], Alan West[2], John Darby[2], Melanie Rees-Roberts[1], Tim Doulton[3], Douglas MacInnes[4], Jamie M O'Driscoll[5], Rachel Borthwick[1], Tracy Pellatt-Higgins[1], Katerina Gousia[1], Vanessa Short[1], Pauline Swift[6], Jonathan Wiles[5]

1. Centre for Health Services Studies, University of Kent, Canterbury, UK,

2. Public Co-applicant, Kent, UK,

3. East Kent Hospitals University NHS Foundation Trust, Canterbury, UK

4. School of Nursing, Midwifery and Social Work Canterbury Christ Church University, Canterbury, Kent, UK

5. School of Psychology and Life Sciences, Canterbury Christ Church University, Canterbury, Kent, UK

6. Epsom and St Helier University Hospitals NHS Trust, Carshalton, UK

Correspondence to Dr Jonathan Wiles jim.wiles@canterbury.ac.uk

For peer review only - http://bmjopen.bmj.com/site/about/guidelines.xhtml

ABSTRACT

The COVID-19 pandemic has significantly impacted upon the delivery of clinical trials in the UK, posing complicated organisational challenges and requiring adaptations; especially to exercise intervention studies based in the community. We aim to identify the challenges of public involvement, recruitment, consent, follow-up, intervention and the healthcare professional (HCP) delivery aspects of a feasibility study of exercise in hypertensive primary care patients during the COVID-19 pandemic. Whilst these challenges elicited many reactive changes which were specific to, and only relevant in the context of 'lockdown' requirements, some of the protocol developments that came about during this unprecedented period have great potential to inform more permanent practices for carrying out this type of research. To this end, we detail the necessary adaptations to many elements of the feasibility study and critically reflect upon our approach to redesigning and amending this ongoing project in order to maintain its viability to date. Some of the more major protocol adaptations, such as moving the study to remote means wherever possible, had further unforeseen and undesirable outcomes (e.g. additional appointments) with regards to extra resources required to deliver the study. However, other changes improved the efficiency of the study, such as the remote informed consent and the direct advertising with pre-screening survey. The adaptations to the study have clear links to the UK Plan for the future of research delivery. It is intended that this specific documentation and critical evaluation will help those planning or delivering similar studies to do so in a more resource efficient and effective way. In conclusion, it is essential to reflect and respond with protocol changes in the current climate in order to deliver clinical research successfully.

For peer review only - http://bmjopen.bmj.com/site/about/guidelines.xhtml

INTRODUCTION

The recent outbreak of coronavirus disease-2019 (COVID-19)[1] and the international response to impose "stay at home" orders resulted in most clinical trials being suspended to recruitment, with the exception of those directly related to the pandemic. In May 2020, the National Institute for Health and Care Research (NIHR) issued guidance for restarting research paused due to COVID-19 for the UK[2] based upon key guiding principles: viability (scientific, clinical, financial or practical reasons), safety, capacity and prioritisation. Whilst these are fundamental to appropriate conduct of clinical trials, it is evident that changes during and following the pandemic present significant organisational challenges.

To help plan and undertake clinical research in the current climate, a structured approach to the redesign of clinical trials is described by Karzai *et al.*[3] who draw attention to eligibility criteria, correlative studies, telehealth and partnerships, with particular emphasis on logistics of clinical trials and suggest that embracing change is vital.

The Medidata group recently identified that data completeness and collection have been a key problem in the pandemic[4] and to mitigate some of the new challenges facing researchers, many regulatory authorities acknowledged the need to allow adaptations to trial recruitment, consent and monitoring[5].

Here, we critically reflect upon our approach to redesigning/amending a feasibility study of the impact of isometric exercise (IE) on arterial hypertension in otherwise healthy adults. This trial involved identification of people with stage 1 hypertension[6], not on anti-hypertensive

For peer review only - http://bmjopen.bmj.com/site/about/guidelines.xhtml

medication and with no relevant co-morbidity. Participants were randomised to a period of isometric/static exercise (IE) and standard care 'lifestyle' advice (SCA) or control (SCA alone). End points included deliverability in the NHS (particularly primary care), fidelity of the intervention and impact on blood pressure (BP)[7].

We estimate that workload to deliver this project increased by >50% with the advent of COVID-19, e.g. the Study Steering Committee needed to meet 3-monthly versus 6-monthly. The physical exercise nature of the trial also brought specific challenges for governance, safety and conduct, including evaluation of participant eligibility and informed consent along with the prescription of IE originally designed to be face-to-face. The study includes physiological measures of fidelity, e.g. BP and heart rate (HR) responses to exercise, and remote monitoring systems needed to be developed for reliable collection of these data. By nature, exercise interventions require ongoing participant motivation[8] and additional methods to support this remotely were required. Because of the reduction in routine and face-to-face follow-up appointments, as well as changes to coding strategy in primary care, fewer patients were identified following searches of GP systems than in pilot work. Indeed, Dale *et al.* suggested that nearly 500,000 fewer people were identified and treated for hypertension in mainland UK from March 2020-2021 compared to the previous year[9]. Paradoxically, it has been reported that the pandemic has heightened the need to focus on lowering the incidence of cardiovascular disease risk factors such as high BP[10]. Whilst physical activity has been identified as a primary focus for cardiovascular disease prevention[11], it is likely that pre-existing barriers to exercise prescription and promotion (e.g. GP perceived status of exercise), have been exacerbated by the pandemic[12]. Recent research suggests that existing reticence amongst GPs based on lack of tradition, as well as lack of

For peer review only - http://bmjopen.bmj.com/site/about/guidelines.xhtml

knowledge and validated tools[13] is likely to have reduced the probability of exercise interventions being implemented. It was therefore necessary to reassess the capacity for NHS primary care staff to deliver the study and ultimately required a fundamental change to recruitment strategies. To help mitigate the impact COVID-19 has caused to research in the NHS, the Department for Health and Social Care (DHSC) launched their strategy regarding the future of UK research delivery[14], recognizing five key themes, three of which are directly addressed in the commentary section of this paper: (2) patient centered, (3) streamlined, efficient and innovative and (4) research enabled by data and digital tools.

We aim to identify the challenges of patient and public involvement, recruitment, consent, follow-up, intervention aspects and primary care staff delivery of a feasibility study of exercise during the COVID-19 pandemic.

COMMENTARY

Patient and public involvement and engagement (PPIE)

Study delivery has benefited from lay members of the project management group, which allowed an integrated approach to redesign. Their previous experience and insight have been invaluable when commenting on important issues, offering a patient perspective to all elements of the redesign including: patient access to technology, use of personal protective equipment, optimising reminder texts (to mitigate attrition) and improving the participant documents and resources. As acknowledged by the NIHR[15], PPIE has been essential in successfully adapting the study for remote delivery during the pandemic and beyond.

For peer review only - http://bmjopen.bmj.com/site/about/guidelines.xhtml

The considerable time delays caused by COVID-19 restrictions along with the numerous amendments, contributed to the significant increase in workload for the research team; arguably this disproportionately impacts upon lay members whose continued involvement is no longer commensurate with initial commitment expectations. Interestingly, similar difficulties have resulted in many COVID-19 trials sacrificing valuable PPIE to meet time constraint pressures[16]. We have been extremely fortunate with the loyalty and commitment received from our public members and would advise anyone embarking on a funded research path to ensure they select these members with care. The importance of careful ongoing consideration of this aspect is reiterated in the UK-wide vision for the future of clinical research delivery which identifies the need to strengthen PPIE in research[14].

Changes to trial protocol and governance

As a result of COVID-19 restrictions, alterations had to be made to the study protocol, along with ethical amendments and this inevitably introduced significant delay to delivery of the study[17]. One major alteration involved moving all contact to remote means wherever possible, including the screening, baseline and follow-up visits. This clearly aligns with the DHSC's fourth key theme to ensure research is enabled by data and digital tools [14]. This meant an additional remote appointment had to be added to screen and check patient eligibility and clinic BP measurements were replaced by participant home BP readings using Omron M3 Intellisense machines resulting in increased study costs. Also, this raised potential concerns regarding the accuracy of using this type of BP monitor[18]: despite the device being validated[19] and the use of remotely observed BP measurements by a trained HCP.

For peer review only

For peer review only - http://bmjopen.bmj.com/site/about/guidelines.xhtml

As a result of social distancing guidelines, participants were asked to carry out home BP readings with the investigator via video call. This was to ensure accurate home BP measurements according to NICE guidelines[6]. The disadvantage of this approach was that participants needed access to technology which PPIE advised to avoid. Implementing this major change in delivery required additional equipment, such as webcam access, instructional resources (e.g. videos), and alternative arrangements for those without IT access or ability i.e. free provision of smart technology or an additional visit. Thus, there were further logistical and cost implications associated with continued attempts to avoid inequity of access.

Due to reduced face-to-face contact with participants, it was necessary to develop a remote reminder system to mitigate increased risk of drop out. The sending of the messages was completely automated and made use of an SMS API provided by a large provider, with this system now reusable for future studies.

Adaptation of participant identification searches and recruitment

The pandemic made recruitment more challenging for several reasons including a reduction of patients identified with stage 1 hypertension on GP records, reduced access to GP administrative staff and less provision of research active staff in primary care.

Searches of GP records yielded considerably fewer patients than pilot work had indicated. This is primarily attributed to: reduced attendances at GP clinics, suspension of routine health checks (e.g., well man over 50), reduced recording of hypertension in primary care (suspension of some indicators in the quality and outcomes framework) and lack of repeat attendance for suspected

For peer review only - http://bmjopen.bmj.com/site/about/guidelines.xhtml

hypertension. This hypothesis is supported by the findings of Dale *et al.*[10], who demonstrate a considerable reduction in numbers treated for incident hypertension during the pandemic. Anecdotal evidence also suggests that because GPs were concerned about being able to follow patients up (during the pandemic), many were commenced on antihypertensive medication immediately following diagnosis rather than allowing a period of lifestyle modification as per hypertension guidelines[6]. Since our study recruited untreated hypertensives, this rendered them ineligible. In future, initiatives such as rollout of the NHS community pharmacy BP check service[20] may mean potential participants for hypertension studies are identified outside the GP setting and supports the need for a more data-enabled research environment[14].

As a result of persistent difficulties with recruiting in primary care, the study was approved for delivery in all NHS settings with additional direct to patient advertising. This targeted potential participants geographically via Facebook social media within reasonable travel distance of a research site. In addition, those displaying interest in subjects that may predispose them to being attracted to the study were targeted. Users seeing the advert could click through to a pre-screening survey to find out if they were eligible to take part in the study and register their details[21]. This led to a greater number of potential participants (75% of those randomised) without involving any NHS staff time. This method also elicited a lower percentage of screen failures compared to GP screening and mail out (31% screen failure rate for direct advertising compared to 67% for mail out). Key learning has been the effectiveness of the pre-screening survey in significantly reducing staff time (up to 12-hours of screening patient lists before mailout) and screen failure rates.

For peer review only - http://bmjopen.bmj.com/site/about/guidelines.xhtml

Adaptations to consent

The requirement to reduce face-to-face contact with participants meant that, although consent remained a requirement, this process had to be managed virtually with the HCP on the video call and participants completing and signing an online form. In line with the drive for research to be enabled by digital tools[14], this data is now captured straight into Qualtrics (online system) which is directly accessible by the research team. This allowed more efficient and accurate data handling without the need to transfer data from paper to database. In general, this worked well, however there were some cases where it did not, primarily because patients were unable to access both Microsoft Teams and Qualtrics simultaneously.

Changes to the intervention - prescription and development of isometric exercise training

The IE intervention used is a wall squat (Figure 1) protocol, which involves leaning against a wall and squatting at an individual specific (knee joint) angle prescribed to elicit the required exercise intensity based upon HR [22].

Figure 1. Isometric wall squat exercise

To accurately prescribe an individual specific wall squat angle, participants must complete at least three-stages of a five-stage incremental isometric exercise test (IIET)[23]. It was originally intended to subjectively pre-assess each patient's physical ability to meet this requirement during the initial face-to-face screening visit. However, since this was replaced with a remote screening visit, it was not possible to complete this capability assessment in person. As such, we had to develop a simple protocol to be completed remotely via video call. This protocol tested the

For peer review only - http://bmjopen.bmj.com/site/about/guidelines.xhtml

participant's ability to reach an approximation of their personalised IE training angle and hold for 60-seconds. The easy-to-follow instructions allow participants to carry out the test independently. Delivering the test remotely required additional risk assessment, the translation of safety considerations into the home (e.g. a nearby chair for support), along with additional online instructional materials.

The IIET stayed the same apart from the time delay of having to establish the status of the exercise type as non-aerosol generating; expert consensus from the Physiological Society was not available until 20th May 2020[24]. However, new personal protective equipment (PPE) considerations had to be implemented immediately in line with government guidelines[25]. This had numerous implications, not least equipment costs and additional time considerations during face-to-face testing.

Impact of COVID-19 on NHS primary care staff participation

It was originally planned to recruit 2-4 primary care sites in the Southeast based upon feasibility searches performed before the pandemic. Between November 2019 and February 2020, one site had committed in principle as a research site. The onset of COVID-19 and unprecedented demands on the NHS, in particular primary care, led to initial difficulties in identifying principal investigators at prospective sites due to uncertainty of workload. Identification of appropriate HCPs with the capacity to deliver the intervention was already a challenge. This was exacerbated by the fact that GP principal investigators were focused on the COVID-19 response and, later, COVID-19 intervention studies and vaccination.

For peer review only - http://bmjopen.bmj.com/site/about/guidelines.xhtml

Embedding clinical research in the NHS is a key theme in the vision of *The Future of UK Clinical Research Delivery*[14]. To create a research-positive culture in which all health and care staff feel empowered to support and participate in clinical research as part of their job role, much greater funding and resourcing of primary care would be necessary. To try and mitigate this in the current study we were forced to approach sites further afield and would strongly recommend over-planning the number of sites in future exercise-based studies.

Conclusions

Delivery of clinical trials in a safe and reliable way has always been complex, requiring good governance and ethical frameworks, as well as robust infrastructure. Whilst there are many randomised controlled trials of exercise either published or planned, their use is more limited than conventional trials of medicinal products. In addition, there are barriers to the prescription of exercise by HCPs. These issues became more acute during the COVID-19 pandemic where, quite reasonably, trials directed at intervention in COVID-19 were prioritised[3]. However, it is evident that abandoning preventative healthcare measures has had (and more concerningly will continue to have) a deleterious effect on the general population.

We have discussed several predictable hurdles the pandemic created for recruitment to a feasibility study of IE. Other unexpected problems have also arisen, such as a significant reduction in the number of people identified with stage 1 hypertension . Ironically, the pandemic presented opportunities such as unprecedented speed and fluidity of change to the study approach. Remote consent and screening of patients, automated reminders, and video validation of BP technique were all developed, approved and tested more rapidly as a result of necessity.

For peer review only - http://bmjopen.bmj.com/site/about/guidelines.xhtml

However, this impacted directly upon our original commitment to ensure equality of access due to the associated IT requirements and level of IT literacy required to engage remotely e.g., need for webcams, two screens open etc. Overall, a willingness to constantly reflect and respond with protocol changes is essential in the current climate.

Since we were unable to identify eligible patients through primary care, we sought and gained approval for direct marketing of the study resulting in a tremendous response (1362 click-throughs from 63 days of active social media advertising), indicating public willingness and enthusiasm for this type of research. Central databases, opt-in to research and direct marketing (where appropriate) are likely to be much more effective methods for future study recruitment.

Finally, it may be worth considering a consensus statement from leaders in the field of exercise research to find common ways to enhance recruitment to trials of exercise to augment current clinical practice.

REFERENCES

1. Huang C, Wang Y, Li X, Ren L, Zhao J, Hu Y, et al. Clinical features of patients infected with 2019 novel coronavirus in Wuhan, China. *Lancet*. 2020; https://doi.org/10.1016/S0140-6736(20)30183-5
2. National Institute for Health and Care Research (NIHR). *Restart Framework - A framework for restarting NIHR research activities which have been paused due to COVID-19*. https://www.nihr.ac.uk/documents/restart-framework/24886?pr= . Accessed 1 February 2021.

For peer review only - http://bmjopen.bmj.com/site/about/guidelines.xhtml

3. Karzai F, Madan R and Dahut W. The World of Clinical Trial Development Post COVID-19: Lessons Learned from a Global Pandemic. *Clinical Cancer Research.* 2020; https://doi.org/10.1158/1078-0432.CCR-20-1914

4. Medidata. *COVID-19 and Clinical Trials: The Medidata Perspective.* https://www.medidata.com/wp-content/uploads/2020/07/COVID19-Response7.0_Clinical-Trials_2020729_v1.pdf . Accessed 22 February 2021.

5. Health Research Authority (HRA). *Making changes to a research study to manage the impact of Covid-19.* https://www.hra.nhs.uk/covid-19-research/covid-19-guidance-sponsors-sites-and-researchers/#. Accessed 1 February 2021.

6. National Institute for Health and Care Excellence (NICE) guideline (NG136). *Hypertension in adults: diagnosis and management*. https://www.nice.org.uk/guidance/ng136/chapter/Recommendations#diagnosing-hypertension. Accessed 21 April 2022.

7. Wiles J, Rees-Roberts M, O'Driscoll J, Doulton T, MacInnes D, Short V et al. Feasibility study to assess the delivery of a novel isometric exercise intervention for people with stage 1 hypertension in the NHS: protocol for the IsoFIT-BP study including amendments to mitigate the risk of COVID-19. *Pilot Feasibility Stud.* 2021; https://doi.org/10.1186/s40814-021-00925-w.

8. Lopes S, Félix G, Mesquita-Bastos J, Figueiredo D, Oliveira J, Ribeiro F. Determinants of exercise adherence and maintenance among patients with hypertension: a narrative review. *Rev Cardiovasc Med*. 2021; https://doi.org/10.31083/j.rcm2204134.

9. Dale CR, Takhar R, Carragher R, Torabi F, Katsoulis M, Duffield S, et al. The adverse impact of COVID-19 pandemic on cardiovascular disease prevention and management in

For peer review only - http://bmjopen.bmj.com/site/about/guidelines.xhtml

England, Scotland and Wales: A population-scale analysis of trends in medication data. *medRxiv* (Preprint) 2022; https://doi.org/10.1101/2021.12.31.21268587.

10. The Lancet Regional Health—Europe. Pandemic heightens the need to combat cardiovascular diseases. *Lancet Reg. Health Eur*. 2021; https://doi.org/10.1016/j.lanepe.2021.100217.
11. Visseren F, Mach F, Smudlers Y, Carbolla D, Koskinas C, Back M., et al. ESC Scientific Document Group, 2021 ESC Guidelines on cardiovascular disease prevention in clinical practice: Developed by the Task Force for cardiovascular disease prevention in clinical practice with representatives of the European Society of Cardiology and 12 medical societies With the special contribution of the European Association of Preventive Cardiology (EAPC). *European Heart Journal*. 2021; https://doi.org/10.1093/eurheartj/ehab484
12. Persson G, Brorsson A, Ekvall Hansson E. et al. Physical activity on prescription (PAP) from the general practitioner's perspective – a qualitative study. *BMC Fam Pract*. 2013. https://doi.org/10.1186/1471-2296-14-128.
13. O'Regan A, Pollock M, D'Sa S, Niranjan V. ABC of prescribing exercise as medicine: a narrative review of the experiences of general practitioners and patients. *BMJ Open Sport & Exercise Medicine.* 2021; http://dx.doi.org/10.1136/bmjsem-2021-001050.
14. Department of Health & Social Care. *The Future of UK Clinical research Delivery.* https://www.gov.uk/government/publications/the-future-of-uk-clinical-research-delivery. Accessed 25 March 2022.
15. National Institute for Health and Care Research. *Different experiences: A framework for considering who might be involved in research.*

For peer review only - http://bmjopen.bmj.com/site/about/guidelines.xhtml

https://www.nihr.ac.uk/documents/different-experiences-a-framework-for-considering-who-might-be-involved-in-research/27387. Accessed 8 June 2021.

16. Health Research Authority (HRA). *Public involvement in a pandemic: lessons from the UK COVID-19 public involvement matching service.* https://www.hra.nhs.uk/planning-and-improving-research/best-practice/public-involvement/public-involvement-pandemic-lessons-uk-covid-19-public-involvement-matching-service/. Accessed 07 June 2021.

17. Health Research Authority (HRA). *Making changes to a research study to manage the impact of COVID-19.* https://www.hra.nhs.uk/covid-19-research/covid-19-guidance-sponsors-sites-and-researchers/. Accessed 07 June 2021.

18. Ruzicka M, Akbari A, Bruketa E, Kayibanda JF, Baril C, Hiremath S. How Accurate Are Home Blood Pressure Devices in Use? A Cross-Sectional Study. *PLoS One*. 2016; https://doi.org/10.1371/journal.pone.0155677

19. Roland A, José K, Jirar T, Feghali E, Ramzi, Mattar J. Validation of Three Automatic Devices for Self-measurement of Blood Pressure According to the International Protocol: the Omron M3 Intellisense (HEM-7051-E), the Omron M2 Compact (HEM 7102-E), and the Omron R3-I Plus (HEM 6022-E). *Blood Pressure Monitoring.* 2010; doi: 10.1097/MBP.0b013e3283354b11.

20. NHS Business Services Authority. *NHS Community Pharmacy Blood Pressure Check Service.* https://www.nhsbsa.nhs.uk/pharmacies-gp-practices-and-appliance-contractors/dispensing-contractors-information/nhs-community-pharmacy-blood-pressure-check-service. Accessed 21 April 2022.

For peer review only - http://bmjopen.bmj.com/site/about/guidelines.xhtml

21. Beauharnais C, Larkin M, Zai A, Boykin E, Luttrell J, Wexler D. Efficacy and cost-effectiveness of an automated screening algorithm in an inpatient clinical trial. *Clinical Trials*. 2012; https://doi.org/10.1177/1740774511434844.

22. Wiles J.D., Goldring N., O'Driscoll J.M., Taylor K. and Coleman D.C. An Alternative Approach to Isometric Exercise Training Prescription for Cardiovascular Health. *Translational Journal of the ACSM*, 3 (2) 2018; doi:10.1249/TJX.0000000000000052

23. Wiles JD, Goldring N, Coleman D. Home-based isometric exercise training induced reductions resting blood pressure. *Eur J Appl Physiol* 2017; https://doi.org/10.1007/s00421-016-3501-0.

24. The Physiological society. *Resuming laboratory testing with human participants*. https://www.physoc.org/covid19/returning-to-the-lab/resuming-laboratory-testing-with-human-participants/. Accessed 21 April 22.

25. UK Health Security Agency. *COVID-19: infection prevention and control (IPC)*. https://www.gov.uk/government/publications/wuhan-novel-coronavirus-infection-prevention-and-control#section-8point1. Accessed 25 March 2022.

AUTHORS' CONTRIBUTIONS

Chris Farmer, Jim Wiles, Melanie Rees-Roberts, Jamie O'Driscoll and Douglas MacInnes designed the original study concept and design. COVID-19 adaptations were implemented by the Chris Farmer and Jim Wiles as co-leads during COVID-19 and learnings collated by Ellie Santer for this paper. The first draft of the manuscript was written by Chris Farmer, Jim Wiles, Ellie Santer and further developed by Melanie Rees-Robers, Alan West, John Darby. The paper was then reviewed by Douglas MacInnes, Vanessa Short, Katerina Gousia, Tracy Pellatt-Higgins,

For peer review only - http://bmjopen.bmj.com/site/about/guidelines.xhtml

Tim Doulton and Jamie O'Driscoll. The study has two lay co-applicants (Alan West and John Darby) who are involved as members of the research team and make shared decisions on the study. Alan West and John Darby directly contributed to drafting and reviewing the content of this manuscript on Patient and Public Involvement during COVID. Pauline Swift is chair of the Study Steering Committee and with Tim Doulton reviewed drafts of this manuscript for clinical aspects within national context of hypertension treatment.

FUNDING STATEMENT

This work was supported by the NIHR research for Patient Benefit (RfPB) Programme (grant number NIHR200485). The views expressed are those of the author(s) and not necessarily those of the NIHR or the Department of Health and Social Care.Additional funding was also granted by Alan Squirrell Artificial Kidney Unit Trust Charity number 254317. **Trial Registration:** ISRCTN, ISRCTN13472393. Registered 9 September 2020, https://doi.org/10.1186/ISRCTN13472393

COMPETING INTERESTS

Pauline Swift has received speaker honorarium from AstraZeneca & Bayer and is a Trustee of the charities Blood Pressure UK and British and Irish Hypertension Society. No conflicts of interest / competing interests were declared by any of the other authors.

For peer review only

For peer review only - http://bmjopen.bmj.com/site/about/guidelines.xhtml

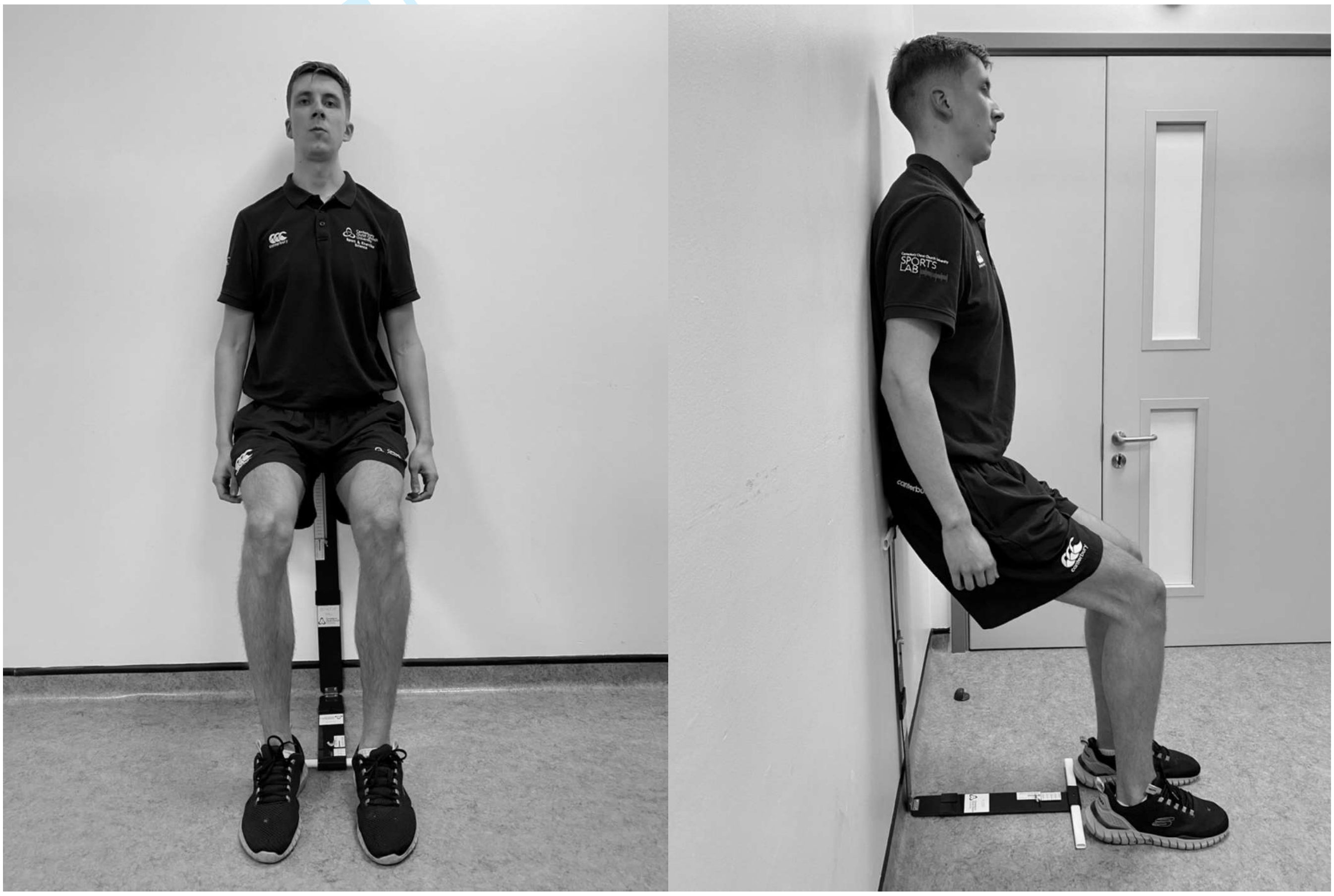

BMJ Open

Delivering clinical studies of exercise in the COVID-19 pandemic: Challenges and adaptations using a feasibility trial of isometric exercise to treat hypertension as an exemplar.

Journal:	*BMJ Open*
Manuscript ID	bmjopen-2022-068204.R2
Article Type:	Communication
Date Submitted by the Author:	06-Mar-2023

Complete List of Authors:	Farmer, C; University of Kent, Centre for Health Services Studies; East Kent Hospitals University NHS Foundation Trust, Renal Department<br>Santer, Ellie; University of Kent, Centre for Health Services Studies<br>West, Alan; Public co-applicant<br>Darby, John; Public co-applicant<br>Rees-Roberts, Melanie; University of Kent, Centre for Health Services Studies<br>Doulton, Timothy; East Kent Hospitals University NHS Foundation Trust, Renal Department<br>MacInnes, Douglas; Canterbury Christ Church Univ, Facult yof Health and Wellbeing<br>O'Driscoll, Jamie; Canterbury Christ Church University, Sport, Exercise and Rehabilitation Sciences<br>Borthwick, Rachel; University of Kent Centre for Health Services Studies<br>Pellatt-Higgins, T; University of Kent, Centre for Health Services studies<br>Gousia, Katerina; University of Kent, Centre for Health Services Studies<br>Short, Vanessa; Newton Place Surgery<br>Swift, Pauline; Epsom and St Helier University Hospitals NHS Trust, Research and Development<br>Wiles, Jonathan; Canterbury Christ Church University, Sport, Exercise and Rehabilitation Sciences

**Primary Subject Heading**:	Communication
Secondary Subject Heading:	Cardiovascular medicine, Evidence based practice, Health services research
Keywords:	Clinical trials < THERAPEUTICS, Hypertension < CARDIOLOGY, SPORTS MEDICINE

SCHOLARONE™
Manuscripts

I, the Submitting Author has the right to grant and does grant on behalf of all authors of the Work (as defined in the below author licence), an exclusive licence and/or a non-exclusive licence for contributions from authors who are: i) UK Crown employees; ii) where BMJ has agreed a CC-BY licence shall apply, and/or iii) in accordance with the terms applicable for US Federal Government officers or employees acting as part of their official duties; on a worldwide, perpetual, irrevocable, royalty-free basis to BMJ Publishing Group Ltd ("BMJ") its licensees and where the relevant Journal is co-owned by BMJ to the co-owners of the Journal, to publish the Work in BMJ Open and any other BMJ products and to exploit all rights, as set out in our licence.

The Submitting Author accepts and understands that any supply made under these terms is made by BMJ to the Submitting Author unless you are acting as an employee on behalf of your employer or a postgraduate student of an affiliated institution which is paying any applicable article publishing charge ("APC") for Open Access articles. Where the Submitting Author wishes to make the Work available on an Open Access basis (and intends to pay the relevant APC), the terms of reuse of such Open Access shall be governed by a Creative Commons licence – details of these licences and which Creative Commons licence will apply to this Work are set out in our licence referred to above.

Other than as permitted in any relevant BMJ Author's Self Archiving Policies, I confirm this Work has not been accepted for publication elsewhere, is not being considered for publication elsewhere and does not duplicate material already published. I confirm all authors consent to publication of this Work and authorise the granting of this licence.

For peer review only

Delivering clinical studies of exercise in the COVID-19 pandemic: Challenges and adaptations using a feasibility trial of isometric exercise to treat hypertension as an exemplar.

Chris Farmer[1], Ellie Santer[1], Alan West[2], John Darby[2], Melanie Rees-Roberts[1], Tim Doulton[3], Douglas MacInnes[4], Jamie M O'Driscoll[5], Rachel Borthwick[1], Tracy Pellatt-Higgins[1], Katerina Gousia[1], Vanessa Short[1], Pauline Swift[6], Jonathan Wiles[5]

1. Centre for Health Services Studies, University of Kent, Canterbury, UK,

2. Public Co-applicant, Kent, UK,

3. East Kent Hospitals University NHS Foundation Trust, Canterbury, UK

4. School of Nursing, Midwifery and Social Work Canterbury Christ Church University, Canterbury, Kent, UK

5. School of Psychology and Life Sciences, Canterbury Christ Church University, Canterbury, Kent, UK

6. Epsom and St Helier University Hospitals NHS Trust, Carshalton, UK

Correspondence to Dr Jonathan Wiles jim.wiles@canterbury.ac.uk

For peer review only - http://bmjopen.bmj.com/site/about/guidelines.xhtml

ABSTRACT

The COVID-19 pandemic has significantly impacted upon the delivery of clinical trials in the UK, posing complicated organisational challenges and requiring adaptations; especially to exercise intervention studies based in the community. We aim to identify the challenges of public involvement, recruitment, consent, follow-up, intervention and the healthcare professional (HCP) delivery aspects of a feasibility study of exercise in hypertensive primary care patients during the COVID-19 pandemic. Whilst these challenges elicited many reactive changes which were specific to, and only relevant in the context of 'lockdown' requirements, some of the protocol developments that came about during this unprecedented period have great potential to inform more permanent practices for carrying out this type of research. To this end, we detail the necessary adaptations to many elements of the feasibility study and critically reflect upon our approach to redesigning and amending this ongoing project in order to maintain its viability to date. Some of the more major protocol adaptations, such as moving the study to remote means wherever possible, had further unforeseen and undesirable outcomes (e.g. additional appointments) with regards to extra resources required to deliver the study. However, other changes improved the efficiency of the study, such as the remote informed consent and the direct advertising with pre-screening survey. The adaptations to the study have clear links to the UK Plan for the future of research delivery. It is intended that this specific documentation and critical evaluation will help those planning or delivering similar studies to do so in a more resource efficient and effective way. In conclusion, it is essential to reflect and respond with protocol changes in the current climate in order to deliver clinical research successfully; as in the case of this particular study.

For peer review only - http://bmjopen.bmj.com/site/about/guidelines.xhtml

Strengths and limitations of the study

- The protocol developments documented provide a useful resource to other researchers and research managers tasked with delivering physical activity / applied research trials in a ‘post covid’ environment.
- The structured approach to the redesign of this clinical trial clearly highlights the advantage of having integrated and comprehensive patient and public involvement
- Recommendations are being made based upon the delivery of a small-scale feasibility study
- The adaptations and implications identified may not be generalisable to all types of study design

INTRODUCTION

The recent outbreak of coronavirus disease-2019 (COVID-19)[1] and the international response to impose “stay at home” orders resulted in most clinical trials being suspended to recruitment, with the exception of those directly related to the pandemic. In May 2020, the National Institute for Health and Care Research (NIHR) issued guidance for restarting research paused due to COVID-19 for the UK[2] based upon key guiding principles: viability (scientific, clinical, financial or practical reasons), safety, capacity and prioritisation. Whilst these are fundamental to appropriate conduct of clinical trials, it is evident that changes during and following the pandemic present significant organisational challenges.

To help plan and undertake clinical research in the current climate, a structured approach to the redesign of clinical trials is described by Karzai *et al.*[3] who draw attention to eligibility criteria,

For peer review only - http://bmjopen.bmj.com/site/about/guidelines.xhtml

correlative studies, telehealth and partnerships, with particular emphasis on logistics of clinical trials and suggest that embracing change is vital.

The Medidata group recently identified that data completeness and collection have been a key problem in the pandemic[4] and to mitigate some of the new challenges facing researchers, many regulatory authorities acknowledged the need to allow adaptations to trial recruitment, consent and monitoring[5].

Here, we critically reflect upon our approach to redesigning/amending a feasibility study of the impact of isometric exercise (IE) on arterial hypertension in otherwise healthy adults. This trial involved identification of people with stage 1 hypertension[6], not on anti-hypertensive medication and with no relevant co-morbidity. Participants were randomised to a period of isometric/static exercise (IE) and standard care 'lifestyle' advice (SCA) or control (SCA alone). End points included deliverability in the NHS (particularly primary care), fidelity of the intervention and impact on blood pressure (BP)[7].

We estimate that workload to deliver this project increased by >50% with the advent of COVID-19, e.g. the Study Steering Committee needed to meet 3-monthly versus 6-monthly. The physical exercise nature of the trial also brought specific challenges for governance, safety and conduct, including evaluation of participant eligibility and informed consent along with the prescription of IE originally designed to be face-to-face. The study includes physiological measures of fidelity, e.g. BP and heart rate (HR) responses to exercise, and remote monitoring systems needed to be developed for reliable collection of these data. By nature, exercise interventions require ongoing

For peer review only - http://bmjopen.bmj.com/site/about/guidelines.xhtml

participant motivation[8] and additional methods to support this remotely were required. Because of the reduction in routine and face-to-face follow-up appointments, as well as changes to coding strategy in primary care, fewer patients were identified following searches of GP systems than in pilot work. Indeed, Dale *et al.* suggested that nearly 500,000 fewer people were identified and treated for hypertension in mainland UK from March 2020-2021 compared to the previous year[9]. Paradoxically, it has been reported that the pandemic has heightened the need to focus on lowering the incidence of cardiovascular disease risk factors such as high BP and obesity [10]. Whilst physical activity has been identified as a primary focus for cardiovascular disease prevention[11], it is likely that pre-existing barriers to exercise prescription and promotion (e.g. GP perceived status of exercise), have been exacerbated by the pandemic[12]. Recent research suggests that existing reticence amongst GPs based on lack of tradition, as well as lack of knowledge and validated tools[13] is likely to have reduced the probability of exercise interventions being implemented. It was therefore necessary to reassess the capacity for NHS primary care staff to deliver the study and ultimately required a fundamental change to recruitment strategies. To help mitigate the impact COVID-19 has caused to research in the NHS, the Department for Health and Social Care (DHSC) launched their strategy regarding the future of UK research delivery[14], recognizing five key themes, three of which are directly addressed in the commentary section of this paper: (2) patient centered, (3) streamlined, efficient and innovative and (4) research enabled by data and digital tools.

We aim to identify the challenges of patient and public involvement, recruitment, consent, follow-up, intervention aspects and primary care staff delivery of a feasibility study of exercise during the COVID-19 pandemic.

For peer review only - http://bmjopen.bmj.com/site/about/guidelines.xhtml

COMMENTARY

Patient and public involvement and engagement (PPIE)

Study delivery has benefited from lay members of the project management group, which allowed an integrated approach to redesign. Their previous experience and insight have been invaluable when commenting on important issues, offering a patient perspective to all elements of the redesign including: patient access to technology, use of personal protective equipment, optimising reminder texts (to mitigate attrition) and improving the participant documents and resources. As acknowledged by the NIHR[15], PPIE has been essential in successfully adapting the study for remote delivery during the pandemic and beyond.

The considerable time delays caused by COVID-19 restrictions along with the numerous amendments, contributed to the significant increase in workload for the research team; arguably this disproportionately impacts upon lay members whose continued involvement is no longer commensurate with initial commitment expectations. Interestingly, similar difficulties have resulted in many COVID-19 trials sacrificing valuable PPIE to meet time constraint pressures[16]. We have been extremely fortunate with the loyalty and commitment received from our public members and would advise anyone embarking on a funded research path to ensure they select these members with care. The importance of careful ongoing consideration of this aspect is reiterated in the UK-wide vision for the future of clinical research delivery which identifies the need to strengthen PPIE in research[14].

Changes to trial protocol and governance

For peer review only - http://bmjopen.bmj.com/site/about/guidelines.xhtml

As a result of COVID-19 restrictions, alterations had to be made to the study protocol, along with ethical amendments and this inevitably introduced significant delay to delivery of the study[17]. One major alteration involved moving all contact to remote means wherever possible, including the screening, baseline and follow-up visits. This clearly aligns with the DHSC's fourth key theme to ensure research is enabled by data and digital tools [14]. This meant an additional remote appointment had to be added to screen and check patient eligibility and clinic BP measurements were replaced by participant home BP readings using Omron M3 Intellisense machines resulting in increased study costs. Also, this raised potential concerns regarding the accuracy of using this type of BP monitor[18]: despite the device being validated[19] and the use of remotely observed BP measurements by a trained HCP.

As a result of social distancing guidelines, participants were asked to carry out home BP readings with the investigator via video call. This was to ensure accurate home BP measurements according to NICE guidelines[6]. The disadvantage of this approach was that participants needed access to technology which PPIE advised to avoid. Implementing this major change in delivery required additional equipment, such as webcam access, instructional resources (e.g. videos), and alternative arrangements for those without IT access or ability i.e. free provision of smart technology or an additional visit. Thus, there were further logistical and cost implications associated with continued attempts to avoid inequity of access.

Due to reduced face-to-face contact with participants, it was necessary to develop a remote reminder system to mitigate increased risk of drop out. The sending of the messages was

For peer review only - http://bmjopen.bmj.com/site/about/guidelines.xhtml

completely automated and made use of an SMS API provided by a large provider, with this system now reusable for future studies.

Adaptation of participant identification searches and recruitment

The pandemic made recruitment more challenging for several reasons including a reduction of patients identified with stage 1 hypertension on GP records, reduced access to GP administrative staff and less provision of research active staff in primary care.

Searches of GP records yielded considerably fewer patients than pilot work had indicated. This is primarily attributed to: reduced attendances at GP clinics, suspension of routine health checks (e.g., well man over 50), reduced recording of hypertension in primary care (suspension of some indicators in the quality and outcomes framework) and lack of repeat attendance for suspected hypertension. This hypothesis is supported by the findings of Dale *et al.*[10], who demonstrate a considerable reduction in numbers treated for incident hypertension during the pandemic. Anecdotal evidence also suggests that because GPs were concerned about being able to follow patients up (during the pandemic), many were commenced on antihypertensive medication immediately following diagnosis rather than allowing a period of lifestyle modification as per hypertension guidelines[6]. Since our study recruited untreated hypertensives, this rendered them ineligible. In future, initiatives such as rollout of the NHS community pharmacy BP check service[20] may mean potential participants for hypertension studies are identified outside the GP setting and supports the need for a more data-enabled research environment[14].

For peer review only

As a result of persistent difficulties with recruiting in primary care, the study was approved for delivery in all NHS settings with additional direct to patient advertising. This targeted potential participants geographically via Facebook social media within reasonable travel distance of a research site. In addition, those displaying interest in subjects that may predispose them to being attracted to the study were targeted. Users seeing the advert could click through to a pre-screening survey to find out if they were eligible to take part in the study and register their details[21]. This led to a greater number of potential participants (75% of those randomised) without involving any NHS staff time. This method also elicited a lower percentage of screen failures compared to GP screening and mail out (31% screen failure rate for direct advertising compared to 67% for mail out). Key learning has been the effectiveness of the pre-screening survey in significantly reducing staff time (up to 12-hours of screening patient lists before mailout) and screen failure rates.

Adaptations to consent

The requirement to reduce face-to-face contact with participants meant that, although consent remained a requirement, this process had to be managed virtually with the HCP on the video call and participants completing and signing an online form. In line with the drive for research to be enabled by digital tools[14], this data is now captured straight into Qualtrics (online system) which is directly accessible by the research team. This allowed more efficient and accurate data handling without the need to transfer data from paper to database. In general, this worked well, however there were some cases where it did not, primarily because patients were unable to access both Microsoft Teams and Qualtrics simultaneously.

For peer review only - http://bmjopen.bmj.com/site/about/guidelines.xhtml

Changes to the intervention - prescription and development of isometric exercise training

The IE intervention used is a wall squat (Figure 1) protocol, which involves leaning against a wall and squatting at an individual specific (knee joint) angle prescribed to elicit the required exercise intensity based upon HR [22].

Figure 1. Isometric wall squat exercise

To accurately prescribe an individual specific wall squat angle, participants must complete at least three-stages of a five-stage incremental isometric exercise test (IIET)[23]. It was originally intended to subjectively pre-assess each patient's physical ability to meet this requirement during the initial face-to-face screening visit. However, since this was replaced with a remote screening visit, it was not possible to complete this capability assessment in person. As such, we had to develop a simple protocol to be completed remotely via video call. This protocol tested the participant's ability to reach an approximation of their personalised IE training angle and hold for 60-seconds. The easy-to-follow instructions allow participants to carry out the test independently. Delivering the test remotely required additional risk assessment, the translation of safety considerations into the home (e.g. a nearby chair for support), along with additional online instructional materials.

The IIET stayed the same apart from the time delay of having to establish the status of the exercise type as non-aerosol generating; expert consensus from the Physiological Society was not available until 20th May 2020[24]. However, new personal protective equipment (PPE) considerations had to be implemented immediately in line with government guidelines[25]. This

For peer review only - http://bmjopen.bmj.com/site/about/guidelines.xhtml

had numerous implications, not least equipment costs and additional time considerations during face-to-face testing.

Impact of COVID-19 on NHS primary care staff participation

It was originally planned to recruit 2-4 primary care sites in the Southeast based upon feasibility searches performed before the pandemic. Between November 2019 and February 2020, one site had committed in principle as a research site. The onset of COVID-19 and unprecedented demands on the NHS, in particular primary care, led to initial difficulties in identifying principal investigators at prospective sites due to uncertainty of workload. Identification of appropriate HCPs with the capacity to deliver the intervention was already a challenge. This was exacerbated by the fact that GP principal investigators were focused on the COVID-19 response and, later, COVID-19 intervention studies and vaccination.

Embedding clinical research in the NHS is a key theme in the vision of *The Future of UK Clinical Research Delivery*[14]. To create a research-positive culture in which all health and care staff feel empowered to support and participate in clinical research as part of their job role, much greater funding and resourcing of primary care would be necessary. To try and mitigate this in the current study we were forced to approach sites further afield and would strongly recommend over-planning the number of sites in future exercise-based studies.

Conclusions

Delivery of clinical trials in a safe and reliable way has always been complex, requiring good governance and ethical frameworks, as well as robust infrastructure. Whilst there are many

For peer review only - http://bmjopen.bmj.com/site/about/guidelines.xhtml

randomised controlled trials of exercise either published or planned, their use is more limited than conventional trials of medicinal products. In addition, there are barriers to the prescription of exercise by HCPs. These issues became more acute during the COVID-19 pandemic where, quite reasonably, trials directed at intervention in COVID-19 were prioritised[3]. However, it is evident that abandoning preventative healthcare measures has had (and more concerningly will continue to have) a deleterious effect on the general population. In context, maintaining healthy lifestyle is important and this itself could be a protective factor during a pandemic like COVID-19 where patients with obesity and other risks factors were affected more.

We have discussed several predictable hurdles the pandemic created for recruitment to a feasibility study of IE. Other unexpected problems have also arisen, such as a significant reduction in the number of people identified with stage 1 hypertension . Ironically, the pandemic presented opportunities such as unprecedented speed and fluidity of change to the study approach. Remote consent and screening of patients, automated reminders, and video validation of BP technique were all developed, approved and tested more rapidly as a result of necessity. However, this impacted directly upon our original commitment to ensure equality of access due to the associated IT requirements and level of IT literacy required to engage remotely e.g., need for webcams, two screens open etc. Overall, a willingness to constantly reflect and respond with protocol changes is essential in the current climate.

Since we were unable to identify eligible patients through primary care, we sought and gained approval for direct marketing of the study resulting in a tremendous response (1362 click-throughs from 63 days of active social media advertising), indicating public willingness and

For peer review only - http://bmjopen.bmj.com/site/about/guidelines.xhtml

enthusiasm for this type of research. Central databases, opt-in to research and direct marketing (where appropriate) are likely to be much more effective methods for future study recruitment.

Finally, it may be worth considering a consensus statement from leaders in the field of exercise research to find common ways to enhance recruitment to trials of exercise to augment current clinical practice.

In closing, whilst this study is still ongoing due to the delays caused by COVID-19, it is evident that we would not have been able to achieve our recruitment targets and the necessary data collection without successfully implementing the changes discussed.

REFERENCES

1. Huang C, Wang Y, Li X, Ren L, Zhao J, Hu Y, et al. Clinical features of patients infected with 2019 novel coronavirus in Wuhan, China. *Lancet*. 2020; https://doi.org/10.1016/S0140-6736(20)30183-5
2. National Institute for Health and Care Research (NIHR). *Restart Framework - A framework for restarting NIHR research activities which have been paused due to COVID-19*. https://www.nihr.ac.uk/documents/restart-framework/24886?pr= . Accessed 1 February 2021.
3. Karzai F, Madan R and Dahut W. The World of Clinical Trial Development Post COVID-19: Lessons Learned from a Global Pandemic. *Clinical Cancer Research.* 2020; https://doi.org/10.1158/1078-0432.CCR-20-1914

For peer review only - http://bmjopen.bmj.com/site/about/guidelines.xhtml

4. Medidata. *COVID-19 and Clinical Trials: The Medidata Perspective.* https://www.medidata.com/wp-content/uploads/2020/07/COVID19-Response7.0_Clinical-Trials_2020729_v1.pdf . Accessed 22 February 2021.

5. Health Research Authority (HRA). *Making changes to a research study to manage the impact of Covid-19.* https://www.hra.nhs.uk/covid-19-research/covid-19-guidance-sponsors-sites-and-researchers/#. Accessed 1 February 2021.

6. National Institute for Health and Care Excellence (NICE) guideline (NG136). *Hypertension in adults: diagnosis and management*. https://www.nice.org.uk/guidance/ng136/chapter/Recommendations#diagnosing-hypertension. Accessed 21 April 2022.

7. Wiles J, Rees-Roberts M, O'Driscoll J, Doulton T, MacInnes D, Short V et al. Feasibility study to assess the delivery of a novel isometric exercise intervention for people with stage 1 hypertension in the NHS: protocol for the IsoFIT-BP study including amendments to mitigate the risk of COVID-19. *Pilot Feasibility Stud.* 2021; https://doi.org/10.1186/s40814-021-00925-w.

8. Lopes S, Félix G, Mesquita-Bastos J, Figueiredo D, Oliveira J, Ribeiro F. Determinants of exercise adherence and maintenance among patients with hypertension: a narrative review. *Rev Cardiovasc Med*. 2021; https://doi.org/10.31083/j.rcm2204134.

9. Dale CR, Takhar R, Carragher R, Torabi F, Katsoulis M, Duffield S, et al. The adverse impact of COVID-19 pandemic on cardiovascular disease prevention and management in England, Scotland and Wales: A population-scale analysis of trends in medication data. *medRxiv* (Preprint) 2022; https://doi.org/10.1101/2021.12.31.21268587.

For peer review only - http://bmjopen.bmj.com/site/about/guidelines.xhtml

10. The Lancet Regional Health—Europe. Pandemic heightens the need to combat cardiovascular diseases. *Lancet Reg. Health Eur*. 2021; https://doi.org/10.1016/j.lanepe.2021.100217.

11. Visseren F, Mach F, Smudlers Y, Carbolla D, Koskinas C, Back M., et al. ESC Scientific Document Group, 2021 ESC Guidelines on cardiovascular disease prevention in clinical practice: Developed by the Task Force for cardiovascular disease prevention in clinical practice with representatives of the European Society of Cardiology and 12 medical societies With the special contribution of the European Association of Preventive Cardiology (EAPC). *European Heart Journal*. 2021; https://doi.org/10.1093/eurheartj/ehab484

12. Persson G, Brorsson A, Ekvall Hansson E. et al. Physical activity on prescription (PAP) from the general practitioner's perspective – a qualitative study. *BMC Fam Pract*. 2013. https://doi.org/10.1186/1471-2296-14-128.

13. O'Regan A, Pollock M, D'Sa S, Niranjan V. ABC of prescribing exercise as medicine: a narrative review of the experiences of general practitioners and patients. *BMJ Open Sport & Exercise Medicine.* 2021; http://dx.doi.org/10.1136/bmjsem-2021-001050.

14. Department of Health & Social Care. *The Future of UK Clinical research Delivery.* https://www.gov.uk/government/publications/the-future-of-uk-clinical-research-delivery. Accessed 25 March 2022.

15. National Institute for Health and Care Research. *Different experiences: A framework for considering who might be involved in research.* https://www.nihr.ac.uk/documents/different-experiences-a-framework-for-considering-who-might-be-involved-in-research/27387. Accessed 8 June 2021.

For peer review only - http://bmjopen.bmj.com/site/about/guidelines.xhtml

16. Health Research Authority (HRA). *Public involvement in a pandemic: lessons from the UK COVID-19 public involvement matching service.* https://www.hra.nhs.uk/planning-and-improving-research/best-practice/public-involvement/public-involvement-pandemic-lessons-uk-covid-19-public-involvement-matching-service/. Accessed 07 June 2021.

17. Health Research Authority (HRA). *Making changes to a research study to manage the impact of COVID-19.* https://www.hra.nhs.uk/covid-19-research/covid-19-guidance-sponsors-sites-and-researchers/. Accessed 07 June 2021.

18. Ruzicka M, Akbari A, Bruketa E, Kayibanda JF, Baril C, Hiremath S. How Accurate Are Home Blood Pressure Devices in Use? A Cross-Sectional Study. *PLoS One*. 2016; https://doi.org/10.1371/journal.pone.0155677

19. Roland A, José K, Jirar T, Feghali E, Ramzi, Mattar J. Validation of Three Automatic Devices for Self-measurement of Blood Pressure According to the International Protocol: the Omron M3 Intellisense (HEM-7051-E), the Omron M2 Compact (HEM 7102-E), and the Omron R3-I Plus (HEM 6022-E). *Blood Pressure Monitoring.* 2010; doi: 10.1097/MBP.0b013e3283354b11.

20. NHS Business Services Authority. *NHS Community Pharmacy Blood Pressure Check Service.* https://www.nhsbsa.nhs.uk/pharmacies-gp-practices-and-appliance-contractors/dispensing-contractors-information/nhs-community-pharmacy-blood-pressure-check-service. Accessed 21 April 2022.

21. Beauharnais C, Larkin M, Zai A, Boykin E, Luttrell J, Wexler D. Efficacy and cost-effectiveness of an automated screening algorithm in an inpatient clinical trial. *Clinical Trials*. 2012; https://doi.org/10.1177/1740774511434844.

For peer review only - http://bmjopen.bmj.com/site/about/guidelines.xhtml

22. Wiles J.D., Goldring N., O'Driscoll J.M., Taylor K. and Coleman D.C. An Alternative Approach to Isometric Exercise Training Prescription for Cardiovascular Health. *Translational Journal of the ACSM*, 3 (2) 2018; doi:10.1249/TJX.0000000000000052

23. Wiles JD, Goldring N, Coleman D. Home-based isometric exercise training induced reductions resting blood pressure. *Eur J Appl Physiol* 2017; https://doi.org/10.1007/s00421-016-3501-0.

24. The Physiological society. *Resuming laboratory testing with human participants*. https://www.physoc.org/covid19/returning-to-the-lab/resuming-laboratory-testing-with-human-participants/. Accessed 21 April 22.

25. UK Health Security Agency. *COVID-19: infection prevention and control (IPC).* https://www.gov.uk/government/publications/wuhan-novel-coronavirus-infection-prevention-and-control#section-8point1. Accessed 25 March 2022.

AUTHORS' CONTRIBUTIONS

Chris Farmer, Jim Wiles, Melanie Rees-Roberts, Jamie O'Driscoll and Douglas MacInnes designed the original study concept and design. COVID-19 adaptations were implemented by Chris Farmer and Jim Wiles as co-leads during COVID-19 and learnings collated by Ellie Santer for this paper. The first draft of the manuscript was written by Chris Farmer, Jim Wiles, Ellie Santer and further developed by Melanie Rees-Robers, Alan West, John Darby. The paper was then reviewed by Douglas MacInnes, Vanessa Short, Katerina Gousia, Tracy Pellatt-Higgins, Rachel Borthwick, Tim Doulton and Jamie O'Driscoll. The study has two lay co-applicants (Alan West and John Darby) who are involved as members of the research team and make shared decisions on the study. Alan West and John Darby directly contributed to drafting and reviewing

For peer review only - http://bmjopen.bmj.com/site/about/guidelines.xhtml

the content of this manuscript on Patient and Public Involvement during COVID. Pauline Swift is chair of the Study Steering Committee and with Tim Doulton reviewed drafts of this manuscript for clinical aspects within national context of hypertension treatment.

FUNDING STATEMENT

This work was supported by the NIHR research for Patient Benefit (RfPB) Programme (grant number NIHR200485). The views expressed are those of the author(s) and not necessarily those of the NIHR or the Department of Health and Social Care. Additional funding was also granted by Alan Squirrell Artificial Kidney Unit Trust Charity number 254317. **Trial Registration:** ISRCTN, ISRCTN13472393. Registered 9 September 2020, https://doi.org/10.1186/ISRCTN13472393

COMPETING INTERESTS

Pauline Swift has received speaker honorarium from AstraZeneca & Bayer and is a Trustee of the charities Blood Pressure UK and British and Irish Hypertension Society. No conflicts of interest / competing interests were declared by any of the other authors.

For peer review only - http://bmjopen.bmj.com/site/about/guidelines.xhtml

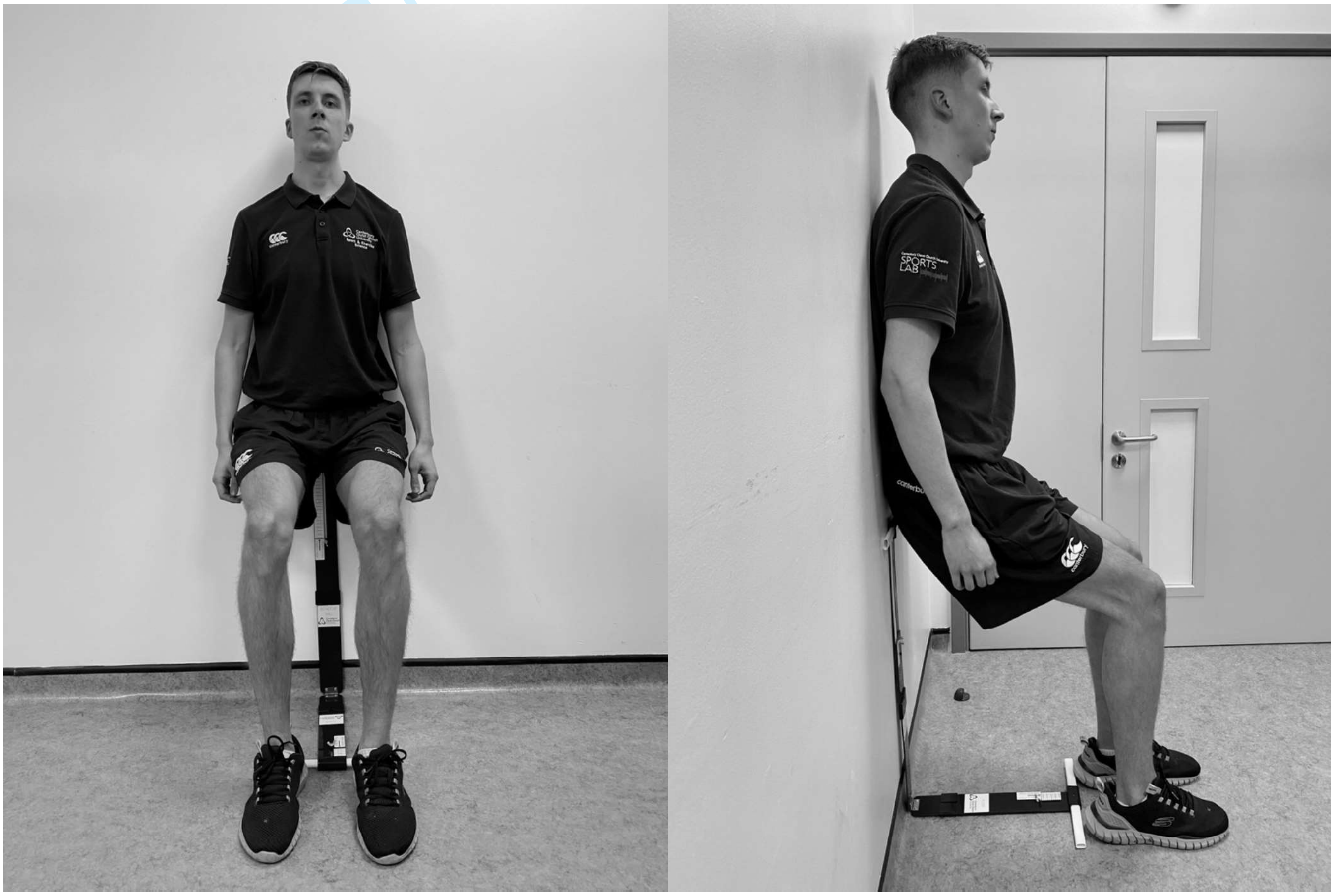
SPORTS
LAB
